# Evaluation of the Impact of Mild Steaming and Heat Treatment on the Concentration of Okadaic Acid, Dinophysistoxin-2 and Dinophysistoxin-3 in Mussels

**DOI:** 10.3390/toxins8060175

**Published:** 2016-06-06

**Authors:** Inés Rodríguez, Amparo Alfonso, Alvaro Antelo, Mercedes Alvarez, Luis M. Botana

**Affiliations:** 1 Departamento de Farmacología, Facultad de Veterinaria, Campus de Lugo, USC, Lugo 27002, Spain; ines.rodriguez@rai.usc.es; 2 Laboratorio CIFGA S.A., Plaza de Santo Domingo 20 5^a^ planta, Lugo 27001, Spain; alvaro@cifga.es (A.A.); mercedes@cifga.es (M.A.)

**Keywords:** steaming, dinophysistoxin, okadaic acid, mass spectrometry

## Abstract

This study explores the effect of laboratory and industrial steaming on mussels with toxin concentrations above and below the legal limit. We used mild conditions for steaming, 100 °C for 5 min in industrial processing, and up to 20 min in small-scale laboratory steaming. Also, we studied the effect of heat on the toxin concentration of mussels obtained from two different locations and the effect of heat on the levels of dinophysistoxins 3 (DTX3) in both the mussel matrix and in pure form (7-O-palmitoyl okadaic ester and 7-O-palmytoleyl okadaic ester). The results show that the loss of water due to steaming was very small with a maximum of 9.5%, that the toxin content remained unchanged with no concentration effect or increase in toxicity, and that dinophysistoxins 3 was hydrolyzed or degraded to a certain extent under heat treatment. The use of liquid-certified matrix showed a 55% decrease of dinophysistoxins 3 after 10 min steaming, and a 50% reduction in total toxicity after treatment with an autoclave (121 °C for 20 min).

## 1. Introduction

In the past years, there has been considerable debate about the influence of heat and cooking on the concentration of marine toxins in shellfish, especially those with a lypophilic nature, and in mussels in particular. This debate was initiated for three reasons. One reason was the description of an important concentration effect of heat or cooking on toxin levels due to the loss of water [1,2]. The second was the demand of canning shellfish producers, since the influence of this heating effect on the toxic levels of a commercial product meant that products that initially contained toxins below the legal regulatory limit of 160 µg/kg flesh [3] would become unsuited for commercialization after heating. The third reason was the change, after January 2015, from mouse bioassay to chromatographic separation with mass spectrometric detection of marine toxins as a reference method for monitoring [4], since the higher detection capabilities of this method as compared to the mouse bioassay would expose the change in concentration after heating.

Initial studies carried out in mussels contaminated with azaspiracids (AZAs) reported that cooking would increase the concentration of azaspiracids twofold [1]. Another study reported later indicated that okadaic acid (OA) and dinophysistoxin-2 (DTX2) would be concentrated after cooking due to a loss of about 50% water, with increases in toxin values of about 30% to 70% in okadaic acid–equivalent concentrations [2].

These conclusions were gathered by the European Food Safety Agengy (EFSA) working group on marine toxins, and they recommended that cooking, steaming and autoclaving should be considered for testing, and the analysis of toxins should be harmonized in order to take into account the changes in toxin concentration [5].

The current protocol for analysis is defined by the EU [4] on the basis of a chromatographic separation with mass spectrometric detection and quantification. The method, given the wide variety of matrices and toxins, is open to fit the technical capabilities of all laboratories, but it has several uncontrolled factors, such as the instrumental technology, a strict need for certified toxin standards, the source and quality of reagents and approaches to perform the analysis such as mass methods, and the toxin solvents and instrument model on toxin quantification, which may render large variations in the final toxin values. We have reported the influence of solvent brands and the use of toxin conversion factors to quantify one toxin using another toxin calibrant [6]. Depending on the different variations in these uncontrolled factors, the error in the quantification can reach 200%.

Steaming and heat sterilization are two of the most frequent manipulations undergone by the shellfish industry. In this article we show the influence of these processes on the toxin concentration. Our conclusion is that for a method with so many potential uncontrolled factors, it is very important to harmonize those prone to the largest variations, especially those related to the values linked to sample weight. Since the final analytical results have to be provided in µg/kg flesh, the processing of flesh during the analysis is critical. We report the influence of steaming and heat sterilization on the final amount of the toxins okadaic acid, dinophysistoxin-2 and dinophysistoxin-3.

## 2. Results and Discussion

### 2.1. Influence of Weight in the Processing of Samples

In order to establish the influence of the weight of the sample on the different steps of the sampling processing, we started by registering the whole weight as the sum of the shell, the water inside the shell and the flesh. We then determined the weight of the water inside the shell, a weight that is lost after opening the valves. According to the protocol, the sample to be taken for extraction, 2 g, must be obtained from a 100 g rinsed flesh homogenate. Therefore, after opening the shell, the flesh was weighed and then it was deposited in a sieve, rinsed with water and weighed again. In the case of steaming mussels, we weighed the amount of flesh and adsorbed water and the weight of the shell.

The mussels were manipulated in groups of 1 kg, and the weighing was repeated three times. The groups were: freshly obtained mussels, mussels steamed in the laboratory (to mimic a domestic cooking process), and mussels steamed in an industrial process (industrial steaming is the process utilized to separate the flesh from the shell). Mussels were from two different sources, Bueu and Riveira. Those from Bueu were large, with about 23 pieces in 1 kg (sample C) and with about 25 pieces in 1 kg (sample A), while mussels from Riveira were smaller, with 35 individuals per kg (sample B).

Table 1 shows the weights of fresh, laboratory-cooked (lab steaming) and industrial-steamed (for shucking) mussels from Riveira, which were small (35 individuals/kg, sample B), and from Bueu, which were large (23 mussels/kg, sample C), both with toxin values below legal limits. We also independently processed 25 large mussels from Bueu with a larger toxin concentration well above legal limits (sample A). In the industrial processing of mussels before steaming, only the initial weight of the whole mussels is known; hence, we could not calculate the percentage of the flesh weight.

The weight data show that, in general, in fresh mussels, the flesh represents between 22.5% ± 0.5% (SD) and 29.2% ± 0.3% (SD) of the total weight, and there is a significant amount of valve water that is lost after opening the mussel which does not contribute to the analysis but which it is considered in the weight of the original product, after debris is removed. We measured weights from 28.4% (Table 1, sample B) to 38.6% (sample C), although very large mussels may have up to 45.9% of valve water (sample C). There is a small valve water loss before the shell is opened; this accounts for 7% (Table 1, Sample 1), 6% (Sample 2), 7.3% (Sample 3) and 6.4% (Sample 4) of the initial weight. In mussels freshly obtained and processed in the factory, the amount of water in the valve is lower, with a value through several months (March to August 2014) of 23.9 ± 6.2 (SD). We can therefore assume that the amount of weight of valve water in fresh mussels accounts for between one-quarter to one-third of the total weight.

### 2.2. Influence of Steaming on Weight

Table 1 shows that after steaming, there is a loss of weight that does not depend on the size of the mussels or on the process, as laboratory steaming and industrial steaming provided very similar results. The maximum loss of weight due to steaming was an extra 9.5% of the initial weight (compared to fresh mussels in sample C, 13% *vs.* 22.5%) when using industrial steaming, but in general steaming accounts for a small loss of weight. Therefore, laboratory steaming does not alter the values when compared to industrial steaming (19% *vs.* 20.6% (sample A), 22.2% *vs.* 21.5% (B) and 13% *vs.* 14.6% (C)). The average weight of steamed flesh compared to the total mussel weight (discarding the valve water weight) in the industrial process from March to August 2014 was 18.2% ± 5.1% (SD). Also, the size of mussels does not modify the result, as small mussels lost 7.7% and 7% weight for laboratory and industrial steaming, respectively, while large mussels lost 7.9% and 9.5%, respectively.

### 2.3. Influence of Steaming on Hydrolysis

The hydrolysis of samples is necessary to calculate the amount of toxins in the form of the esters that may render OA, DTX1 or DTX2. This is necessary because DTX3 is not toxic, but its hydrolysis in the digestive system releases the active toxins that may cause intoxications [7]. The samples used contained mainly OA esters, which is a typical toxin profile for Galician coasts with *Dynophisis acuminata* and *Dynophisis acuta* [8,9,10]. The samples steamed in the industry were boiled for 2 min at 105 °C and then another 3 min at 100 °C. Also, the samples steamed in the laboratory mimicked a domestic cooking process, with water boiling for 10 min in a 5 L cooking pot with 100 mL of water. The results after hydrolysis show that mussels accumulate a rather important amount of DTX3 in the form of esters of OA and DTX2, although the largest amount of DTX3 corresponds to OA, with DTX2 esters being a maximum of 10% of the total DTX3 in the case of fresh mussels from the location of Riveira (Table 2). There is a clear difference with regard to the source of mussels. The samples taken from Bueu contain no DTX2 in the form of esters. The percent of OA esters is also different depending on the location due to different exposure levels and the species of microalgae causing the bloom, with values that go from 38% to 82%. It is interesting that the percentage of esterified OA or DTX2 is different in the same sample, such as in the 35 fresh mussels sampled from Riveira, that show 81.6% and 48.6% esterified toxin for OA and DTX2, respectively. With regard to the initial toxin value, the highest increase in percent of OA or DTX2 after hydrolysis is 427.6% and 101.5%, respectively, in fresh product (sample B, Table 2). This is attributable to mussels located in different places of the farming area, probably with different feeding and algae exposure conditions. The values for DTX2 are close to the limit of quantification (LOQ) (6.24 µg/kg), and therefore must be taken with caution.

In general, steaming increases the amount of toxins per kg (Table 2), as in all cases the amount of OA/kg is higher after laboratory steaming and even higher after industrial steaming, the increases being 332 < 467 < 670, 14 < 20 < 21, and 80 < 171 < 187 for the levels of OA in the three samples. Total toxin shows similar values in all cases before and after steaming, although there is a slight decrease of total toxin after steaming in small mussels from Riveira, and a slight increase after steaming in large mussels from Bueu. The logical conclusion is that this increase in toxicity is caused by conversion of DTX3 to unesterified acid. In the case of mussels from Riveira, there is a slight decrease of total toxin.

It is a remarkable observation that in the case of the 1 kg sample from Bueu, fresh mussels evolved after steaming from below legal limits to above legal limits (160 μg of OA equivalents/kg), but this is attributable to a variation in the analytical results in different samples, as this was not observed in samples from Riveira, or in the set of 25 mussels with a higher concentration from Bueu.

### 2.4. Influence of Steaming and Autoclaving on DTX3 Levels with no Loss of Water

Table 2 shows the effect of steaming on the concentration of DTX3. The results show that although the total toxins remains the same, the amount of esterified toxins is greatly decreased, with the values being 203 > 35 > 0, 62 > 38 > 19, and 60 = 76 > 0 for the levels of OA-DTX3 in the three samples. Also, it is clear that the steaming in the industrial process is more aggressive to DTX3 than laboratory steaming. This destruction of DTX3 with heat may be related to the presence of the flesh. We have reported in a previous article that high pressure combined with an acidic pH can destroy OA [11]. Since sterilization combines pressure with heat, we decided to compare the effect of steaming or autoclave treatment on DTX3 concentration. As a consequence, we studied the effect of sterilization (121 °C and 1.1 atm) in known amounts of DTX3 in a liquid-certified matrix that was not sterilized during the preparation process, using sealed vials to prevent any water loss by evaporation (Table 3A). Also, we studied the effect of steaming in homogenous mussel tissue (Mussel_Control) with DTX3 levels in sealed vials. In the case of homogenous mussel tissue (Mussel_Control), we did not study the autoclave effect, since the preparation of the matrix involves a sterilization process, hence the matrix was already modified (Table 3B).

There is a 59.4% decrease in the levels of DTX3 in the liquid-certified matrix (Mussel-DSP-2) after steaming for 10 min (Table 3A). The results also show that sterilization does not decrease the level of DTX3 compared to the effect of steaming. Also, 20 min steaming does not further modify the levels of DTX3. On the contrary, steaming does not modify the levels of DTX3 in homogenous mussel tissue (Mussel_Control) (Table 3B), but these results have to be taken with caution, as this matrix was treated with a sterilization process during the preparation. Taking into account the effect shown in Table 3A, this process may have already decreased the original level of DTX3 to a minimum.

Although the changes in the DTX3 levels are quite clear in liquid matrix after 10 min steaming (Table 3A), the total DTX3 does not change (669 µg/kg *vs.* 539 µg/kg), but there is a rather significant decrease (about 50%) of total toxicity after the autoclave treatment (369 µg/kg *vs.* 197 µg/kg or 669 µg/kg *vs.* 327 µg/kg). These results are therefore in agreement with previous studies that reported a decrease in concentration after heat treatment for 10 min and 100 °C to 150 °C [2], although we show a higher decrease of total toxicity after the autoclave treatment, and a slight toxicity reduction after steaming.

### 2.5. Influence of Matrix and Steaming on Pure DTX3 Stability with No Loss of Water

To understand the effect of heat in DTX3 stability, we used three pure DTX3 compounds, namely A (7-O-palmitoyl okadaic ester), B (7-O-palmytoleyl okadaic ester) and C (mixture of isomers A and B). The same amount was added to 2 mL of water, or 2 g of mussel tissue (Mussel_Control). These solutions were cooked for 10 min and 20 min in sealed vials to avoid water loss (control was at time 0). Although we did not know the initial amount of DTX3 analogues, we estimated the value after hydrolysis by measuring OA. The results, shown in Table 4, indicate that pure DTX3 compound A is slightly degraded after steaming for 20 min (561 > 530 > 403 µg/kg), and compound B is further degraded by heat (535 > 460 > 338 µg/kg). A surprising result was that the mixture of esters in sample C was not modified by hydrolysis, which seems to suggest the toxin values were below the quantitation limit for sample C. The addition of the compounds to homogenous mussel tissue (Mussel_Control) seems to provide an environment which makes the compounds resistant to heat, as there is no decrease of the toxins. The conclusion to this is that the presence of matrix seems to stabilize DTX3 and makes the analogues more resistant to heat treatment.

**Figure 1:**
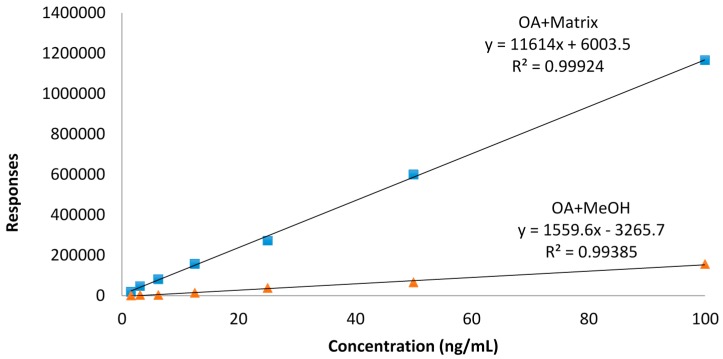
Calibration curves for Okadic Acid (OA) in MeOH and matrix in the range 1.56–100 ng/mL.

Our data are rather consistent in terms of toxin values, showing a decrease of toxins with higher temperatures. Although there are reports that describe unusual matrix effects that cause an increase in toxin concentrations of both OA, DTX2 [12] or AZA3 [13], we have not observed any of these effects, which rules out matrix effects other than those eliminated by the calibration procedure, as shown in Figure 1.

The overall conclusion to our data is that DTX3 in seafood is destroyed by heat under steaming conditions, and that this destruction depends on the matrix environment. Sterilization does pose a stability issue to the toxins, as steaming treatment means a reduction in the amount of DTX3, but not a reduction in total toxicity, since OA or DTX2 are thermostable, but not at 121 °C, with a 50% decrease in total toxicity. The loss of water has been reported as a source of higher toxicity due to the increase of the concentration of toxins in the matrix [1,2], but our data indicates that this loss of water, once the water contained in the valves is discarded, is not higher than 7.8%. Our results do not sustain the observation reported earlier of a 30% water loss after steaming [12]. The difference is attributable to the stronger conditions used, 130 °C for 70 s, while we used milder conditions, 105 °C for 120 s and then 100 °C for 180 s.

Under the mild industrial conditions we studied, it is clear that water loss is very small and that toxicity is unaltered, even though we show that DTX3 is destroyed at a certain level by heat, and largely (50%) by sterilization. Another technical aspect to bear in mind, although we have not studied it, is the reduction of water content after frying the flesh for the canning industry, where a 20% loss of water has to be added to the steaming process.

## 3. Conclusions

In this study, we demonstrate that DTX3 in seafood is destroyed by heat under steaming conditions and that this destruction depends on the matrix environment. Although the total toxicity does not decrease, the sterilization is a stability issue for the toxins and steaming decreases the amount of DTX3. The total toxicity decrease is 50% since OA and DTX2 are thermostable, but not at 121 °C. Taking into account the loss of water, once the water contained in the valves is discarded, it is not higher than 7.8%, and the toxicity is unaltered, even though we show that DTX3 is destroyed to a certain degree by heat, and largely (50%) by sterilization.

## 4. Methods

### 4.1. Reagents

Mussels were extracted in October and November 2014, and they were randomly used with regard to sex, using the same sampling and collection channels as used to reach the market.

Pure DTX2, DTX3 and OA were purchased from Laboratorios CIFGA S.A. (Lugo, Spain). Ampoules contained 0.5 mL of solution with 3.03 µmol DTX2 ± 0.2/kg and 30.13 ± 2.2 µmol OA/kg.

Quality Control Standard Mussel matrix with DSP toxins (Mussel-DSP-2) was purchased from Laboratorios CIFGA S.A. (Lugo, Spain). The Mussel-DSP-2 contained 4 g with 361 ± 34 µg OA/kg, 206 ± 19 µg DTX1/kg and 283 ± 54 µg DTX3/kg, and 8 g of homogenate tissue of mussel (Mussel_Control) with 34.6 ± 4 µg OA/kg and 29.8 ± 3 µg DTX3/kg.

Acetonitrile and methanol were supplied by Panreac (Barcelona, Spain). All solvents employed in this work were HPLC or analytical grade and the water was distilled and passed through a water purification system (Milli-Q, Millipore, Madrid, Spain). Formic acid was purchased from Merck (Darmstadt, Germany). Ammonium formate was from Fluka (Sigma-eAldrich, Madrid, Spain).

### 4.2. Matrix Effect

The homogenate tissue of mussel was spiked with different amounts of okadaic acid to obtain a calibration line. The results were compared against a methanolic calibration line without matrix. The calibration concentration points used were in the range 1.56 to 100 ng/mL and 1.56 ng/mL to 50 ng/mL and no deviations in higher concentrations and in the amount of toxin calculated were reported (Figure 1). The final matrix effect was estimated to be about 2.5 times higher than the methanolic calibration line. The effect was especially high with the highest concentrations. In each analysis, a methanolic and a spiked matrix calibration line were reproduced for calibration purposes.

### 4.3. Recovery

The recovery was calculated with three different experiments:

- Two mussel matrices of known concentration were used, a matrix of certified reference control mussels and a mussel tissue homogenate. The mussel matrices were extracted using the procedure described in the harmonized Standard Operating Procedure (SOP) published by the EU-RL-MB [14].
- The homogenate tissue of mussel was spiked with a known quantity of OA (75 ng/mL final concentration). The matrix was extracted using the procedure described in the EU-harmonized SOP published by the EU-RL-MB [14].
- Three mussel samples were extracted twice with methanol 100% following the procedure described in the EU-harmonized SOP published by the EU-RL-MB [14].

In cases a and b, the LC-MS results gave us similar concentration, 99% ± 2% of accuracy.

In the case c, three samples were extracted twice by the procedure and were measured each extraction separately by LC-MS. In the second extraction 0 ng/mL of OA were detected, in conclusion all OA of the samples was extracted in the first extraction.

### 4.4. Dinophysistoxin-3

Premarketed standards of dinophysistoxins-3 kindly provided by Laboratorios CIFGA S.L. (Lugo, Spain) were used. Since DTX3 toxins are acyl ester, several fatty acids can sterify the molecule of okadaic acid, and we used three different esters, named compounds A (7-O-palmitoyl okadaic ester) 96.3% purity, B (7-O-palmytoleyl okadaic ester) 99.5% purity and C (mixture of isomers of A and B).

### 4.5. Steaming

For this process, along with the samples steamed in the factory (2 min at 105 °C and then another 3 min at 100 °C), we utilized a process to resemble a domestic cooking process, that was done in a container with lid with 100 mL water in the bottom. Boiling water was kept for 10 min. After this process, all parts, and the water in the bottom were weighted.

### 4.6. Sterilization (Heat)

Three samples of quality controlled mussel matrix with DSP toxins were sterilized at 121 °C, 1.1 bar of pressure for 20 min in an autoclave (AE-75-DRY, Raypa, R. Espinar S.L., Barcelona, Spain).

### 4.7. Extraction and Hydrolysis Procedure

Samples were extracted following the EU-Harmonized Standard Operating Procedure for determination of lipophilic marine biotoxins in mollusks by LC-MS/MS published by the EU-RL-MB in January 2015 [14]. First 2.00 g ± 0.05 g of tissue homogenate was weighted into a centrifuge tube. Then 9 mL of methanol was added and the sample was homogenized via vortex mixing for 3 min at maximum speed level. Then, the samples were centrifuged (1010 × g 10 min) at 20 °C and the supernatant was transferred to a 20 mL volumetric flask. The extraction was repeated with the residual pellet with another 9 mL of methanol using a high speed homogenizer (T25 digital Ultra-Turrax, IKA-Werke GmbH & Co. KG, Staufen, Germany) for 1 min. After centrifugation (3700 rpm × 10 min) at 20 °C, the supernatant was transferred and combined. The final volume was made up to 20 mL with methanol. The determination of free okadaic acid (OA), pectenotoxins (PTXs), azaspiracids (AZAs) and yessotoxins (YTXs) was performed by LC-MS/MS after filtering an aliquot of this methanolic extract (0.45 µm filter).

In order to detect and quantify the total content of OA and DTXs, 2.5 mL of methanolic extract were hydrolyzed with 313 µL of 2.5 M NaOH, the mixture was homogenized and heated at 76 °C for 40 min. It was then cooled at room temperature, neutralized with 313 µL of 2.5 M HCl and homogenized in the vortex. The extract was filtered with 0.45 µm filter and then analyzed.

### 4.8. LC-MS/MS Analysis

Chromatographic separation was carried out using a 1290 Infinity ultra-high-performance liquid chromatography system coupled to a 6460 Triple Quadrupole mass spectrometer (Agilent Technologies, Waldbronn, Germany). The toxins were separated using a column AQUITY UPLC BEH C18 (2.1 × 100 mm, 1.7 µm, Waters) at 40 °C. Mobile phase A was 100% water and B acetonitrile-water (95:5), both containing 50 mM formic acid and 2 mM ammonium formate. The gradient program with a flow rate of 0.4 mL/min was started with 30% B and then a linear gradient to 70% B in 3 min. After an isocratic hold time linear of 1.5 min at 70% B and return to the starting conditions of 30% B in 0.1 min. Finally, 30% B was kept for 1.99 min before the next injection. The samples in the autosampler were cooled to 4 °C and the injection volume was 5 µL [8].

MS detection was performed using an Agilent G6460C triple quadrupole mass spectrometer equipped with an Agilent Jet Stream ESI source (Agilent Technologies, Waldbronn, Germany). Source conditions were optimized to achieve the best sensitivity for all compounds: 350 °C of drying gas temperature with 8 L/min flow, nebulizer gas pressure of 45 psi (Nitrocraft NCLC/MS from Air Liquid), sheath gas temperature of 400 °C and a flow of 11 L/min. The capillary voltage was set to 4000 V in negative mode with a nozzle voltage of 0 V and 3500 V in positive mode with a nozzle voltage of 500 V. The fragmentor was 260 V and the cell accelerator voltage was 4. The collision energy was optimized using MassHunter Optimizer software for each toxin (Table 5). For the calibration curve, seven different concentrations of the standard (Cifga, Lugo, Spain) were injected in triplicate: OA/DTX1/DTX2 from 1.56 ng/mL to 100 ng/mL. All toxins were quantified, using their peak areas to calculate amounts and using the curve obtained from each standard. The LOD and LOQ for OA/DTX1/DTX2 were 0.47 ng/mL (1.96 µg/kg) and 1.56 ng/mL (6.24 µg/kg), respectively.

## Figures and Tables

**Table:** (**A:** Weight distribution in the samples)

Product Conditions	Sample A: 25 Large Mussels (Bueu)				Sample B: 35 Mussels (Riveira)				Sample C: 23 Mussels (Bueu)			
	Whole (W) (g)	Flesh (g)	%	Valve Water (mL) % of W	Whole (W) (g)	Flesh (g)	%	Valve Water (mL) % of W	Whole (W) (g)	Flesh (g)	%	Valve Water (mL) % of W
Fresh	1176 ± 29.5	299.6 ± 6.2	25.5% ± 0.2	367.4 ± 13.5 31.2%	880 ± 43.1	257 ± 12.4	29.2% ± 0.3	250 ± 16.6 28.4%	1004 ± 26.3	226 ± 7.3	22.5% ± 0.5	388 ± 4.4 38.6%
Lab Steaming	1140 ± 54.2	234 ± 9.9	20.6% ± 0.4	350 ± 2.8 30.7%	903.9 ± 46.3	195 ± 11.7	21.5% ± 0.2	283 ± 16.6 31.3%	986 ± 48.8	143 ± 6.5	14.6% ± 0.3	453 ± 43.5 45.9%
Industrial Steaming *	-	279 ± 6.2	19% ± 1 (#)	-	-	301 ± 8.1	22.2% (&)	-	-	184 ± 2.2	13% (**)	-

**Table:** (**B:** Weight distribution in starting product)

Sample	Fresh Mussels from Farm with Debris and Valve Water (g)	Debris Removed (g)	Valve Water (mL)	Number of Mussels
Sample 1	5659	311	400	109
Sample 2	6690	581	400	122
Sample 3	7577	635	550	215
Sample 4	7846.5	672	500	142

* Weight loss of the industrial steamed product: (#) 5.63 kg (93 mussels) renders 1050 g of flesh (18%) and 5.53 kg (92 mussels) renders 1138 g of flesh (20%); (&) 10 kg of mussels renders 2218.9 g (22.2%) and (**) 10 kg of mussels renders 1298.1 g (13%) 2.2. Influence of steaming on weight.

**Table 2:** Effect of hydrolysis on Okadaic Acid (OA) and Dinophysistoxin-2 (DTX2) and Dinophysistoxin-3 (DTX3) concentration before and after steaming. There are two samples from Bueu, with very large and small mussels, and one sample from another location (Riveira).

Product Conditions	Hydrolysis	Sample A	Sample B	Sample C
**Fresh Product**	Before (µg/kg OA/DTX2)	332 ± 11/104 ± 5.5	14.5 ± 1.9/6.7 ± 1.4	80 ± 7.6/29.2 ± 1.3
	After (µg/kg OA/ DTX2)	535 ± 36.5/89 ± 2.4	76 ± 3.3/13.5 ± 1.5	140 ± 19.8/23 ± 0.4
	DTX3 (µg/kg OA-ester/DTX2-ester)	203/nd	62/6.8	60/nd
	% Increased toxin above initial value	61.14/nd	427.6/101.5	75/nd
**Steamed in the Laboratory**	Before (µg/kg OA/DTX2)	467 ± 16.9/108 ± 5.2	19.9 ± 0.6/12.7 ± 2.1	171 ± 10.4/33 ± 2.7
	After (µg/kg OA/ DTX2)	502 ± 23.2/77.5 ± 3.5	58 ± 4.25/11.8 ± 1.6	247 ± 13/24.6 ± 2.6
	DTX3 (µg/kg OA-ester/DTX2-ester)	35/nd	38/nd	76/nd
	% Increased toxin above initial value	7.4/nd	190.9/nd	44.4/nd
**Steamed in the Industry**	Before (µg/kg OA/DTX2)	669.9 ± 33.1/152 ± 2.5	20.7 ± 1.3/7.3 ± 1.5	187 ± 18.7/51 ± 2.7
	After (µg/kg OA/ DTX2)	588 ± 41/93.6 ± 7	39.6 ± 2.6/10 ± 2.5	162 ± 12.2/30 ± 2
	DTX3 (µg/kg OA-ester/DTX2-ester)	nd/nd	18.9/2.7	nd/nd
	% Increased toxin above initial value	nd/nd	91.3/36.9	nd/nd

* nd = not detected.

**Table 3:** Effect of steaming on okadaic acid, dinophysistoxin 2 and dinophysistoxin 3 levels. (**A:** Standar matrix Mussel-DSP-2) DTX3 hydrolysis in liquid quality control standard matrix (Mussel-DSP-2) with 361 ± 34 µg/kg AO, 206 ± 19 DTX1, 283 ± 54 DTX3; pH 5.17. Steaming for 0, 10 and 20 min; (**B:** Standar matrix Mussel_Control) DTX3 hydrolysis in homogenous mussel tissue (Mussel_Control) reference material with 34.6 ± 4 µg/kg OA, 29.8 ± 3 µg/kg DTX3. Steaming for 0, 10 and 20 min.

**A:** Standar matrix Mussel-DSP-2				
**Hydrolysis**	**at Time 0**	**at 10 min**	**at 20 min**	After Autoclave *
Before hydrolysis (µg/kg OA)	369 ± 33	417 ± 35	442 ± 38	197 ± 18
After hydrolysis (µg/kg OA)	669 ± 59	539 ± 46	570 ± 49	327 ± 13
OA ester (µg/kg OA)	300	122	128	130
**B:** Standar matrix Mussel_Control				
**Hydrolysis**	**at Time 0**	**at 10 min**	**at 20 min**	After Autoclave **
Before hydrolysis (µg/kg OA)	39 ± 14	38 ± 11	48 ± 13	-
After hydrolysis (µg/kg OA)	70 ± 28	73 ± 18	70 ± 18	-
OA ester (µg/kg OA)	31	35	22	-

* This liquid matrix was not sterilized in the preparation process and contains 0.05 g/mL oxitretracycline; ** This matrix was previously sterilized (121 °C for 30 min) in the preparation process, and contains 0.02 oxotetracycline.

**Table 4:** Effect of hydrolysis on three types of pure DTX3 in water or spiked in homogenous mussel tissue (with 34.6 ± 4 µg/kg AO, 29.8 ± 3 µg/kg DTX3), and performed in a sealed vial with no water lose during cooking. All samples were spiked with the same volume of an unknown quantity of DTX3 with a purity of 96% (A), 99.5 (B), and a pure mixture of three esters, 44% (C16:1-OA), 24% (C16:0-OA), 32% (C16:0-OA). Water indicates addition of 5 µL A, 50 µL B and 50 µL C to 2 mL water. Matrix indicates addition of 5 µL A, 50 µL B and 50 µL C to 2 g of homogenous mussel tissue (Mussel_Control). Steaming for 0, 10 and 20 min.

**DTX3 (µg/kg OA)**	**7-O-palmitoyl Okadaic Ester**					
	**A**	**A**	**A**	**A**	**A**	**A**
Time	Control (0’)	Control (0’)	10´	10´	20´	20´
	Water	Matrix	Water	Matrix	Water	Matrix
Before hydrolysis	0	35 ± 8	0	20 ± 6	0	22 ± 6
After hydrolysis	561 ± 73	114 ± 16	530 ± 69	116 ± 17	403.7 ± 54	110 ± 16
**DTX3 (µg/kg OA)**	**7-O-palmytoleyl Okadaic Ester**					
	**B**	**B**	**B**	**B**	**B**	**B**
Time	Control (0’)	Control (0’)	10´	10´	20´	20´
	Water	Matrix	Water	Matrix	Water	Matrix
Before hydrolysis	0	31 ± 7	0	36 ± 8	0	28 ± 7
After hydrolysis	536 ± 70	145 ± 20	460 ± 61	199 ± 26	338 ± 47	179 ± 24
**DTX3 (µg/kg OA)**	**Mixture of A and B**					
	**C**	**C**	**C**	**C**	**C**	**C**
Time	Control (0’)	Control (0’)	10´	10´	20´	20´
	Water	Matrix	Water	Matrix	Water	Matrix
Before hydrolysis	0	33 ± 7	0	32 ± 8	0	27 ± 7
After hydrolysis	0	104 ± 7	0	96 ± 6	0	87 ± 6

**Table 5:** Mass spectrometric parameters used for the identification of each toxin.

Compound Name	Precursor Ion	Product Ion	Collision Energy	Polarity
DTX1	817.5	255.2	53	Negative
	817.5	113	66	-
OA/DTX2	803.5	255.2	52	Negative
	803.5	113.2	60	-
